# The relationship between tumour necrosis, systemic inflammation, body composition and survival in patients with colon cancer

**DOI:** 10.1038/s44276-024-00119-w

**Published:** 2025-02-05

**Authors:** Ross D. Dolan, Kathryn Pennel, Joshua Thompson, Molly McKenzie, Peter Alexander, Colin Richards, Douglas Black, Tanvir Abbass, Noori Maka, Josh McGovern, Antonia Roseweir, Stephen T. McSorley, Paul G. Horgan, Campbell Roxburgh, Donald C. McMillan, Joanne Edwards

**Affiliations:** 1 Academic Unit of Surgery, School of Medicine, University of Glasgow, Glasgow Royal Infirmary, Glasgow, UK; 2 School of Cancer Sciences, University of Glasgow, Glasgow, UK

## Abstract

**Background:**

In cancer cachexia the relationship between the tumour, its environment and the systemic inflammatory response is not clear. This study aims to examine this relationship in greater detail.

**Methods:**

Host characteristics included the presence of a Systemic Inflammatory Response (SIR) as measured by Systemic Inflammatory Grade (SIG), sarcopenia (SMI) and myosteatosis (SMD) were measured. Categorical variables were analysed using χ^2^ test for linear-by-linear association, or χ^2^ test for 2 by 2 tables. Survival analysis was carried out using univariate and multivariate Cox regression.

**Results:**

A total of 473 patients were included. Of these, 70.4% were over 65 years of age, 54.8% were male and 49.8% had an ASA grade of 1 or 2. Pathological examination showed that the majority of patients had a T3 (53.7%) or a T4 (34.0%) cancer and 73.0% had evidence of necrosis. A SIG score of 0 or 1 was present in 57.7% of patients. Tumour necrosis was associated with age (*p* < 0.01), tumour location (*p* < 0.01), T-stage (*p* < 0.001), margin involvement (*p* < 0.05), SIG (*p* < 0.001), SMI (*p* < 0.01), SMD (*p* < 0.05) and 5-year survival (*p* < 0.001). On multivariate survival analysis in patients with T3 cancers age (HR: 1.45 95% CI 1.13–1.86 *p* < 0.01), ASA grade (HR: 1.50 95% CI 1.15–1.95 *p* < 0.01) and SIG (HR: 1.28 95% CI 1.11–1.48 *p* < 0.001) remained independently associated with survival.

**Conclusion:**

These results suggest that tumour necrosis and the subsequent SIR could result in profound changes in body composition and survival. Further pre-clinical and clinical work is required to prove causation.

## Background

The mechanism by which a tumour brings about profound changes in host metabolism and body composition has long been discussed, especially as the tumour constitutes such a small percentage of host tissue [1, 2]. In the last decade it has become clear that, in addition to the aggressiveness of the tumour, local and systemic inflammatory responses are key to the cachectic decline of the cancer patient [3–5]. However, to date there are no specific inflammatory pathways that have been targeted and proven to reverse the cachectic process and so the current interest is in multimodal interventions for its treatment [4, 6]. Although there is no definitive anti-inflammatory treatment for cancer cachexia, this may be due to previous studies not selecting patients for anti-inflammatory treatment on the basis of their inflammatory status [7] and our lack of understanding of the tumour host inflammatory responses.

Cachexia is generally thought of as disease related malnutrition with increasing importance placed upon systemic inflammation [8]. Recently, the Global Leadership Initiative on Malnutrition (GLIM) have developed standardised diagnostic criteria for malnutrition [9]. This consists of three phenotypic criteria (involuntary weight loss, low BMI, reduced muscle mass) and two aetiologic criteria (reduced food intake and inflammation or disease burden). The definition of cachexia is not currently fixed as can be seen by the recent cachexia endpoints review series [10–14]. However, it is generally accepted that a clinical diagnosis of cachexia has one phenotypic and one aetiological factor (outlined above).

Primary operable colorectal cancer is a useful model to examine such interactions since approximately 40% of patients are systemically inflamed and are sarcopenic (hallmarks of cachexia, GLIM criteria) and the tumour is resected providing the opportunity for detailed tumour analysis. It is now recognised from previous work with tumour invasiveness (T-stage) and necrosis that some aspects of the local and systemic inflammatory response are perturbed. Furthermore, T stage was significantly associated with lower Klintrup–Makinen (KM) grade, lower Immunoscore, higher tumour stroma percentage, elevated modified Glasgow Prognostic Score (mGPS) and Neutrophil Lymphocyte Ratio (NLR) [15, 16].

Richards and coworkers [17], in 343 patients with primary operable colorectal cancer, reported that there were significant associations between tumour necrosis and T-stage, anaemia, white cell count, mGPS and local inflammatory cell infiltrate [17]. Guthrie and coworkers [18], in 118 patients with primary operable colorectal cancer, reported that when normalised for T stage, tumour necrosis was significantly associated with interleukin-6, interleukin-10, vascular endothelial growth factor (VEGF), modified Glasgow Prognostic Score (mGPS), neutrophil–lymphocyte ratio (NLR), white cell, neutrophil, and platelet counts, and skeletal muscle index [18]. More recently, in >1000 patients with colorectal cancer, Kastinen and coworkers reported that tumour necrosis was significantly directly associated with the Glasgow Microenvironment score (GMS a combination tumour inflammatory cell infiltrate and tumour stroma summarised in Table 2), serum levels of interleukin-6 and CXCL8 and inversely associated with mesenteric serum levels of CXCL10 and mast cell densities in the invasive margin of the tumour [19]. Therefore, there is a continuing interest in how the interaction between tumour necrosis and the local and systemic inflammatory responses forms a unifying mechanism by which the tumour microenvironment results in a systemic metabolic upset. The aim of the present study was to examine the relationship between tumour necrosis, the systemic inflammatory response, body composition and outcomes in patients with colon cancer.

## Methods

### Patients

**Fig. 1:**
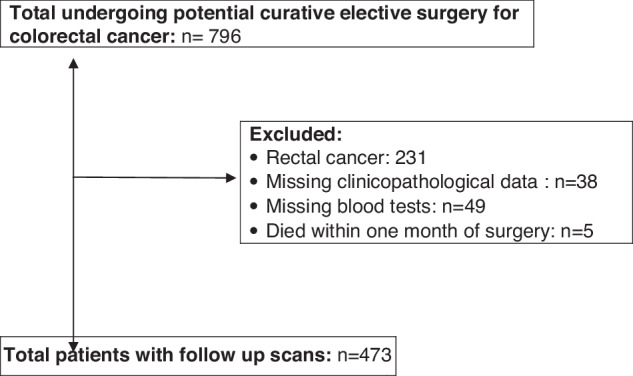
CONSORT flow diagram of patients excluded from the study and final numbers included.

Consecutive patients who underwent elective, potentially curative resection for colon cancer between March 2008 and June 2013 at a single centre were identified from a prospectively maintained database. Those patients with a preoperative CT scan and a recorded height and weight were included. Patients excluded from the study are summarized in Fig. 1.

Patients were classified according to Body Mass Index (BMI) as underweight (BMI < 18.5), normal weight (BMI 18.5–24.9), overweight (BMI 25.0–29.9) and obese (BMI > 30). All tumours were staged according to TNM 5^th^ edition [20]. Preoperative haematological and biochemical markers were recorded.

Nutritional status was assessed using the Malnutrition Universal Screening Tool (MUST) which is included as a part of admission checklist prior to commencing oncology treatment and is performed by admitting nursing staff. MUST is a bed side assessment of patient weight loss, BMI and nutritional intake [21]. Using MUST, patients were classified into low (MUST = 0, *n* = 114), medium (MUST = 1, *n* = 12) and high malnutrition risk (MUST ≥ 2, *n* = 18).

The cause and date of death were confirmed with the Registrar General (Scotland) until 1st June 2017 which served as the censor date. Consent for inclusion of patient data in our prospectively maintained database including CT image analysis was taken as part of the pre-operative consent process. Those with metastatic disease and those who underwent emergency surgery or palliative surgery were excluded from the study. The majority of patients had T3 disease therefore, further sub-analysis was carried out on patients with T3 disease to ascertain if the effects of tumour necrosis were independent of T3-stage. Ethical approval was granted by the West of Scotland Research Ethics Committee, Glasgow (WS/16/0207).

### CT derived analysis

CT images were obtained at the level of the third lumbar vertebra as previously described [13]. Patients whose scans were taken 3 months or more prior to their surgery were excluded from the study. Scans with significant movement artefact or missing region of interest were not considered for inclusion. Each image was analysed using a free-ware program (NIH Image J version 1.47, http://rsbweb.nih.gov/ij/**)** shown to provide reliable measurements [14].

Region of interest (ROI) measurements were made of visceral fat (VFA), subcutaneous fat (SFA), and skeletal muscle areas (SMA) (cm^2^) using standard Hounsfield Unit (HU) ranges (adipose tissue −190 to −30, and skeletal muscle −29 to +150) [22]. These were then normalised for height [2] to create indices; subcutaneous fat index (SFI, cm^2^/m^2^), and skeletal muscle index (SMI, cm^2^/m^2^). Skeletal muscle radiodensity (SMD, HU) was measured from the same ROI used to calculate SMI, as its mean HU. The thresholds used for defining high sub cutaneous fat, visceral fat, sarcopenia and myosteatosis are described previously and summarised in Table 1.

**Table 1:** CT derived body composition measures and thresholds used and Systemic Inflammatory Grade (SIG) Calculation.

Body composition measurement	
High SFI [17]:	
Males > 50.0 cm^2^m^2^ and Females > 42.0 cm^2^m^2^	
Visceral obesity [20, 21]:	
VFA : Males >160 cm^2^ and Females >80 cm^2^	
Sarcopenia	
SMI (Martin) [21]:	
Males: BMI ≤ 25 kg/m^2^ and SMI < 43 cm^2^m^2^ or BMI > 25 kg/m^2^ and SMI < 53 cm^2^m^2^	
Females: BMI ≤ 25 kg/m^2^ and SMI < 41 cm^2^m^2^ or BMI > 25 kg/m^2^ and SMI < 41 cm^2^m^2^	
SMI (Dolan BMI > 25) [1]:	
Males: BMI ≤ 25 kg/m^2^ and SMI < 45 cm^2^m^2^ or BMI > 25 kg/m^2^ and SMI < 53 cm^2^m^2^	
Females: BMI ≤ 25 kg/m^2^ and SMI < 39 cm^2^m^2^ or BMI > 25 kg/m^2^ and SMI < 41 cm^2^m^2^	
Myosteatosis	
SMD (Martin) [21]:	
BMI ≤ 25 kg/m^2^ and SMD < 41 HU or BMI > 25 kg/m^2^ and SMD < 33HU	
SMD (Dolan BMI > 25) [1]:	
BMI ≤ 25 kg/m^2^ and SMD < 34 HU or BMI > 25 kg/m^2^ and SMD < 32HU	
SIG: Systemic Inflammatory Grade:	
mGPS	Score
C-reactive protein ≤ 10	0
C-reactive protein > 10 and albumin ≥ 35	1
C-reactive protein > 10 and albumin < 35	2
NLR	
NLR < 3	0
NLR 3–5	1
NLR > 5	2
Systemic Inflammatory Grade (SIG)	
SIG 0	
mGPS 0 and NLR < 3	
SIG 1	
mGPS 0 and NLR 3–5 or mGPS 1 and NLR < 3	
SIG 2	
mGPS 0 and NLR > 5 or mGPS 2 and NLR < 3 or mGPS 1 and NLR 3–5	
SIG 3	
mGPS 1 and NLR > 5 or mGPS 2 and NLR 3–5	
SIG 4	
mGPS 2 and NLR > 5	

*SFI* Subcutaneous Fat Index (subcutaneous obesity), *VFA* Visceral Fat Area (visceral obesity), *SMI* Skeletal Muscle Index (sarcopenia), *SMD* Skeletal Muscle Density (myosteatosis), *mGPS* modified Glasgow Prognostic Score, *NLR* Neutrophil Lymphocyte Ratio, *SIG* Systemic Inflammatory Grade.

Measurements were performed by two individuals (RD) and (SM) and inter-rater reliability was assessed in a sample of 30 patient images using inter-class correlation coefficients (ICCC) (TFA ICCC = 1.000, SFA ICCC = 1.000, VFA ICCC = 1.000, SMA ICCC = 0.998, SMD ICCC = 0.972). Investigators were blind to patient’s demographic and clinico-pathological status.

An autoanalyzer was used to measure serum CRP (mg/L) and albumin (g/L) concentrations in addition to differential white blood cell counts (Architect; Abbot Diagnostics, Maidenhead, UK). The modified Glasgow Prognostic Score (mGPS) and Neutrophil Lymphocyte Ration (NLR) were derived as previously described [15]. These were then used to calculate the Systemic Inflammatory Grade (SIG) as outlined in Table 1.

### Tissue phenotyping

**Fig. 2:**
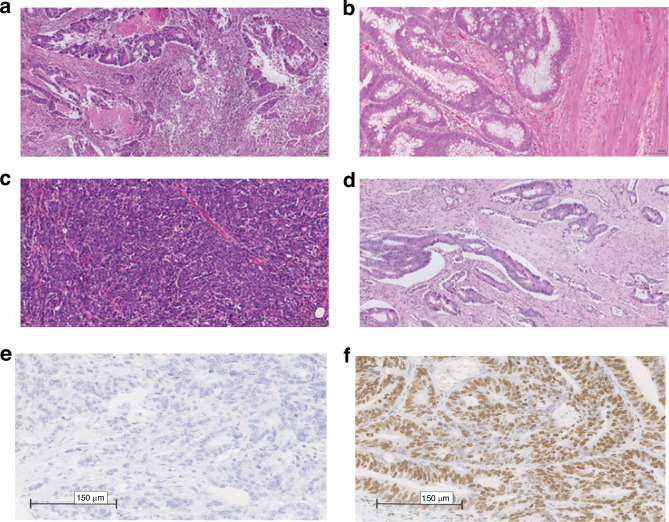
Assessment of tumour inflammatory cell infiltrate and TSP on H&E-stained sections. **a** high KM grade. **b** low KM grade. **c** low TSP. **d** high TSP. **e** Low Ki67 and **f** High Ki67 [25].

Phenotypic subtyping was carried out in pulled tumour blocks as part of the formation of the TMA from samples collected at the time of surgical resection. Four phenotypic characteristics were examined: tumour necrosis; Ki67 proliferation index, Klintrup–Makinen (KM) grade for inflammatory infiltrate and stromal invasion using tumour stroma percentage (TSP) (Fig. 2).

Tumour necrosis was assessed using the method described by Pollheimer et al. [23]. Briefly, at X40 magnification, the full sections were examined for evidence of tumour necrosis. Tumour necrosis was graded as ‘absent’ (none), ‘focal’ (<10% of tumour surface area), ‘moderate’ (10–30% tumour surface area), or ‘extensive’ (>30% of tumour surface area) in each section before an assessment of overall extent of necrosis was made. To test the reliability of the evaluation of necrosis, sections of 30 patients (average of 3 slides per patient) were examined independently by two observers (RD and MM) blinded to clinical outcome and clinicopathological variables. The intraclass correlation coefficient (ICC) for the assessment of tumour necrosis was 0.80.

Immunohistochemical analysis for Ki67 was performed using established protocols from the Institute of Cancer Sciences with appropriate positive and negative controls. Dako anti-Ki-67 (monoclonal mouse anti-human, Ki-67 antigen, clone MIB1, code M7240, DAKO, Glostrup, Denmark) was used at dilution 1: 50 overnight at 4C for immunohistochemistry and was visualised using IMPRESS detection system (Vector Laboratories). Slides were counterstained with haematoxylin, dehydrated and mounted with DPX. Stained slides were scanned using a Hamamatsu NanoZoomer (Hertfordshire, UK). Visualisation and automated cell counts were carried out using the Slidepath Tissue IA system version 3.0 (SlidePath’s Tissue IA system, Dublin, Ireland). Ki67 was graded as low if ≤30% positive cells and high if >30% positive cells [24].

KM grade was assessed by examining immune cell density at the invasive margin on H&E full sections of the tumour taken at the deepest point of invasion. TSP was carried out using H&E-stained full sections taken at the deepest point of invasion.

### Scoring the glasgow microenvironment score

H&E-stained whole sections representing the deepest point of invasion were scored manually for GMS using NDP view software (Hamamatsu). Slides were first scanned onto the server with Hamamatsu NanoZoomer at x20 magnification (Welwyn Garden City, UK). Firstly Klintrup–Mäkinen Grade (KM) and Tumour Stroma Percentage (TSP) were scored as previously described [25]. Briefly, KM was scored semi-quantitatively at the tumour’s invasive margin as weak (either no inflammatory cells or mild increase only) or strong (a band or cup-like infiltrate of inflammatory cells present with associated evidence of tumour nest destruction). Tumour stromal percentage was assessed by estimating the percentage area occupied by stroma vs tumour, to the nearest 10%, in the tumour’s centre at ×100 magnification, not counting areas of mucin or necrosis. TSP was dichotomised into low stroma (≤50%) or high stroma (>50%). GMS scores were then combined as follows: strong KM with any TSP scored GMS 0; weak KM with low TSP scored GMS 1; weak KM with high TSP scored GMS 2 (Table 2).

**Table 2:** GMS Score summary for patients undergoing surgery for colorectal cancer.

	KM Grade	TSP
GMS 0	Strong	Any
GMS 1	Weak	Low
GMS 2	Weak	High

*GMS* Glasgow Microenvironment Score, *KM* Klintrup–Mäkinen Grade, *TSP* Tumour Stroma Percentage.

### Statistical analysis

Body composition measurements were presented as median and range and compared using Mann–Whitney or Kruskal–Wallis tests. Categorical variables were analysed using χ2 test for linear-by-linear association, or χ2 test for 2 by 2 tables. Five year overall survival rates were examined using a life table approach and results were displayed as the percentage of 5-year survival and on survival analysis, statistical significance was calculated using the log-rank test.

Mortality within 30 days of the index procedure or during the index admission were excluded from subsequent survival analysis. The time between the date of surgery and the date of death of any cause was used to define overall survival (OS). Survival data were analysed using univariate and multivariate Cox regression in patients with T3 disease. Those variables associated with at least 200 observations and a of *P* < 0.05 were entered into a backward conditional multivariate model.

Missing data were excluded from analysis on a variable by variable basis. Two tailed *p* values < 0.05 were considered statistically significant. Statistical analysis was performed using SPSS software (Version 21.0. SPSS Inc., Chicago, IL, USA).

## Results

A total of 473 patients were included of these 70.4% were over 65 years of age, 54.8% were male and 49.8% had an ASA grade of 1 or 2. Pathological examination showed that the majority of patients had a tumour breaching the muscularis propria with 53.7% being T3 and 34.0% being T4 respectively. Venous invasion was seen in 50.7% of resected tumours with 28.5% showing tumour budding and 5.9% showing margin involvement. In excess of 10% of tumour necrosis was seen in 48.4% of resected cancers and a GMS score of 0 or 1 was present in 78.3% of patients. Adjuvant therapy was used in 24.6% of cases. A SIG score of 0 or 1 was present in 57.7% of patients.

Body composition showed a BMI ≤ 25 in 68.5% of patients. Subcutaneous obesity as defined by Ebadi et al. [26] was present in 82.9% of patients. Visceral obesity as defined by Doyle et al. [27] was present in 73.5% of patients. Sarcopenia as defined by Martin et al. [22] and Dolan et al. [28] were present in 52.5% and 59.1% of patients respectively. Myosteatosis as defined by Martin et al. [22] and Dolan et al. [28] were present in 63.5% and 58.8% of patients respectively.

Tumour necrosis was associated with age (*p* = 0.002), tumour location (*p* = 0.003), T-stage (*p* < 0.001), margin involvement (*p* = 0.017), SIG (*p* < 0.001), SMI (Martin, *p* = 0.003), SMI (Dolan, *p* = 0.009), SMD (Dolan, *p* = 0.017) and 5-year survival (*p* < 0.001) (Table 3). In patients with T3 cancers tumour necrosis was associated with age (*p* = 0.020), margin involvement (*p* = 0.009), SIG (*p* = 0.003), SMI (Dolan, *p* = 0.045) and 5 year survival (*p* < 0.001) (Table 4).

**Table 3:** The relationship between tumour necrosis, clinicopathological characteristics, tumour phenotyping and CT derived body composition in patients undergoing surgery for colon cancer (*n* = 473).

Characteristic					
		<10% Necrosis	10–30% Necrosis	>30% Necrosis	*P*-value
(*n* = 473)		(*n* = 244)	(*n* = 120)	(*n* = 109)	
Age	≤65	80 (32.8)	36 (30.0)	24 (22.0)	0.002
	65–74	85 (34.8)	42 (35.0)	28 (25.7)	
	>74	79 (32.4)	42 (35.0)	57 (52.3)	
Sex	Female	106 (43.4)	48 (40.0)	60 (55.0)	0.088
	Male	138 (56.6)	72 (60.0)	49 (45.0)	
ASA score (*n* = 408)	1	28 (11.5)	8 (6.7)	13 (11.9)	0.336
	2	83 (34.0)	46 (38.3)	25 (20.8)	
	3	91 (37.3)	42 (35.0)	48 (44.4)	
	4	12 (5.9)	7 (5.8)	5 (45.8)	
Tumour location	Right	122 (50.0)	64 (53.3)	74 (67.9)	0.003
	Left	122 (50.0)	56 (46.7)	35 (32.1)	
T-stage	1	14 (5.7)	2 (1.7)	0 (0.0)	<0.001
	2	34 (13.9)	5 (4.2)	3 (2.8)	
	3	126 (51.6)	65 (54.2)	63 (57.8)	
	4	70 (28.7)	48 (40.0)	43 (39.4)	
N-stage	0	161 (66.0)	68 (56.7)	65 (59.6)	0.346
	1	60 (24.6)	33 (27.5)	36 (33.0)	
	2	23 (9.4)	19 (15.8)	8 (7.3)	
Ki-67 (*n* = 419)	>30	139 (65.3)	86 (71.7)	69 (72.6)	0.092
	<30	74 (34.7)	25 (20.8)	26 (27.4)	
Tumour budding (*n* = 456)	<10	170 (71.4)	72 (60.0)	79 (76.0)	0.675
	>10	68 (28.6)	42 (35.0)	25 (24.0)	
Venous invasion	No	126 (51.6)	51 (42.5)	56 (51.4)	0.693
	Yes	118 (48.3)	69 (57.5)	53 (48.6)	
Margin involvement	No	234 (96.0)	114 (95.0)	97 (89.0)	0.017
	Yes	10 (4.1)	6 (5.0)	12 (11.0)	
MMR status (*n* = 459)	dMMR	39 (16.7)	27 (22.7)	24 (22.6)	0.586
	pMMR	155 (66.2)	74 (62.2)	60 (56.6)	
	MLH1/PMS2 or MHS 2/6	40 (17.1)	18 (15.1)	22 (20.8)	
GMS (*n* = 460)	0	44 (18.6)	16 (13.8)	13 (12.0)	0.176
	1	144 (61.0)	72 (62.1)	71 (65.7)	
	2	48 (20.3)	28 (24.1)	24 (22.2)	
Adjuvant chemotherapy	No	187 (77.0)	87 (72.5)	82 (75.2)	0.607
	Yes	56 (23.0)	33 (27.5)	27 (24.8)	
SIG	0	85 (34.8)	38 (31.7)	28 (25.7)	<0.001
	1	68 (27.9)	29 (24.2)	25 (22.9)	
	2	57 (23.4)	29 (24.2)	21 (19.3)	
	3	28 (11.5)	15 (12.5)	19 (17.4)	
	4	6 (2.5)	9 (7.5)	16 (14.7)	
	Nutrition Assessment				
MUST (*n* = 144)	Low Risk	73 (83.0)	25 (75.8)	16 (69.6)	0.189
	Medium Risk	5 (5.7)	5 (15.2)	2 (8.7)	
	High Risk	10 (11.4)	3 (9.1)	5 (21.7)	
	Body composition				
BMI (kg/m^2^) (*n* = 181)	≤25	33 (29.5)	13 (31.0)	11 (40.7)	0.304
	>25	79 (70.5)	29 (69.0)	16 (59.3)	
High SFI (*n* = 181)	No	20 (17.9)	6 (14.3)	5 (18.5)	0.906
	Yes	92 (82.1)	36 (85.7)	22 (81.5)	
Visceral obesity (*n* = 181)	No	29 (25.9)	8 (19.0)	11 (40.7)	0.303
	Yes	83 (74.1)	34 (81.0)	16 (59.3)	
Low SMI (sarcopenia) (*n* = 181)					
SMI (Martin) (*n* = 181)	No	62 (55.4)	17 (40.5)	7 (25.9)	0.003
	Yes	50 (44.6)	25 (59.5)	20 (74.1)	
SMI (Dolan BMI > 25) (*n* = 181)	No	59 (52.7)	17 (40.5)	7 (25.9)	0.009
	Yes	53 (47.3)	25 (59.5)	20 (74.1)	
Low SMD (Myosteatosis) (*n* = 148)					
SMD (Martin) (*n* = 148)	No	37 (41.1)	12 (35.3)	5 (20.8)	0.075
	Yes	53 (58.9)	22 (64.7)	19 (79.2)	
SMD (Dolan BMI > 25) (*n* = 148)	No	42 (46.7)	15 (44.1)	4 (16.7)	0.017
	Yes	48 (53.3)	19 (55.9)	20 (83.3)	
5 Year survival %(SE)		178 (73.0)	70 (58.3)	56 (51.4)	<0.001

*MMR* Mismatch Repair, *GMS* Glasgow Microenvironment Score, *SIG* Systemic Inflammatory Grade, *MUST* Malnutrition Universal Screening Tool, *BMI* Body Mass Index, *SFI* Subcutaneous Fat Index (subcutaneous obesity), *VFA* Visceral Fat Area (visceral obesity), *SMI* Skeletal Muscle Index (sarcopenia), *SMD* Skeletal Muscle Density (myosteatosis).

**Table 4:** The relationship between tumour necrosis, clinicopathological characteristics, tumour phenotyping and CT derived body composition in patients undergoing surgery for T3 stage colon cancer (*n* = 254).

Characteristic					
		<10% necrosis	10–30% necrosis	>30% necrosis	*P*-value
*n* = 254		(*n* = 126)	(*n* = 65)	(*n* = 63)	
Age	≤65	38 (30.2)	19 (29.2)	13 (20.6)	0.020
	65–74	44 (34.9)	18 (27.7)	15 (23.8)	
	>74	44 (34.9)	28 (43.1)	35 (55.6)	
Sex	Female	53 (42.1)	24 (36.9)	31 (49.2)	0.463
	Male	73 (57.9)	41 (63.1)	32 (50.8)	
ASA score (*n* = 217)	1	12 (10.8)	5 (9.1)	8 (15.7)	0.071
	2	44 (39.6)	22 (40.0)	10 (19.6)	
	3	50 (45.0)	24 (43.6)	29 (56.9)	
	4	5 (4.5)	4 (7.3)	4 (7.8)	
Tumour location	Right	68 (54.0)	37 (56.9)	42 (66.7)	0.108
	Left	58 (46.0)	28 (43.1)	21 (33.3)	
N-stage	0	83 (65.9)	43 (66.2)	37 (58.7)	0.632
	1	32 (25.4)	15 (23.1)	22 (34.9)	
	2	11 (8.7)	7 (10.8)	4 (6.3)	
Ki67 (*n* = 223)	<30%	77 (70.6)	43 (72.9)	40 (72.7)	0.753
	>30%	32 (29.4)	16 (27.1)	15 (27.3)	
Tumour budding (*n* = 244)	<10	89 (71.8)	42 (67.7)	47 (81.0)	0.281
	>10	35 (28.2)	20 (32.3)	11 (19.0)	
Venous invasion	No	67 (53.2)	32 (49.2)	39 (61.9)	0.343
	Yes	59 (46.8)	33 (50.8)	24 (38.1)	
MMR status (*n* = 204)	dMMR	19 (15.6)	16 (24.6)	17 (27.4)	0.119
	pMMR	79 (64.8)	39 (60.0)	34 (54.8)	
	MLH1/PMS2 or MHS 2/6	24 (19.7)	10 (15.4)	11 (17.7)	
GMS (*n* = 247)	0	24 (19.8)	12 (18.8)	7 (11.3)	0.232
	1	76 (62.8)	42 (65.6)	42 (67.7)	
	2	21 (17.4)	10 (15.6)	13 (21.0)	
Adjuvant chemotherapy	No	98 (77.8)	50 (76.9)	49 (77.8)	0.980
	Yes	28 (22.2)	15 (23.1)	14 (22.2)	
Margin involvement	No	126 (100.0)	65 (100.0)	60 (95.2)	0.009
	Yes	0 (0.0)	0 (0.0)	3 (4.8)	
SIG	0	45 (35.7)	22 (33.8)	14 (22.2)	0.003
	1	33 (26.2)	15 (23.1)	16 (25.4)	
	2	30 (23.8)	18 (27.7)	13 (20.6)	
	3	16 (12.7)	5 (7.7)	10 (15.9)	
	4	2 (1.6)	5 (7.7)	10 (15.9)	
	Nutrition assessment				
MUST (*n* = 76)	Low risk	36 (85.7)	18 (75.0)	7 (70.0)	0.329
	Medium risk	1 (2.4)	4 (16.7)	1 (10.0)	
	High risk	5 (11.9)	2 (8.3)	2 (20.0)	
	Body composition				
BMI (kg/m^2^) (*n* = 95)	≤25	36 (67.9)	20 (69.0)	10 (76.9)	0.581
	>25	17 (32.1)	9 (31.0)	3 (23.1)	
High SFI (*n* = 95)	No	11 (20.8)	5 (17.2)	2 (15.4)	0.607
	Yes	42 (79.2)	24 (82.8)	11 (84.6)	
Visceral obesity (*n* = 95)	No	14 (26.4)	5 (17.2)	5 (38.5)	0.718
	Yes	39 (73.6)	24 (82.8)	8 (61.5)	
Low SMI (sarcopenia)					
SMI (Martin) (*n* = 95)	No	25 (47.2)	12 (41.4)	2 (15.4)	0.058
	Yes	28 (52.8)	17 (58.6)	11 (84.6)	
SMI (Dolan BMI > 25) (*n* = 95)	No	23 (43.4)	12 (41.4)	1 (7.7)	0.045
	Yes	30 (56.6)	17 (58.6)	12 (92.3)	
Low SMD (myosteatosis) (*n* = 79)					
SMD (Martin) (*n* = 79)	No	14 (32.6)	8 (32.0)	2 (18.2)	0.442
	Yes	29 (67.4)	17 (68.0)	9 (81.8)	
SMD (Dolan BMI > 25) (*n* = 79)	No	18 (41.9)	9 (36.0)	2 (18.2)	0.171
	Yes	25 (58.1)	16 (64.0)	9 (81.8)	
5 Year survival %(SE)		99 (78.6)	40 (61.5)	32 (50.8)	<0.001

*MMR* Mismatch Repair, *GMS* Glasgow Microenvironment Score, *SIG* Systemic Inflammatory Grade, *MUST* Malnutrition Universal Screening Tool, *BMI* Body Mass Index, *SFI* Subcutaneous Fat Index (subcutaneous obesity), *VFA* Visceral Fat Area (visceral obesity), *SMI* Skeletal Muscle Index (sarcopenia), *SMD* Skeletal Muscle Density (myosteatosis).

On univariate survival analysis in patients with T3 cancers age (*p* < 0.001), ASA (*p* < 0.001), adjuvant therapy (*p* = 0.001), necrosis (*p* = 0.045), SIG (*p* < 0.001), MUST (*p* < 0.001) Sub cutaneous obesity (*p* = 0.006) and Sarcopenia (Dolan, *p* = 0.038) were independently associated with overall survival (Table 5). On multivariate survival analysis age (HR: 1.45 95% CI 1.13–1.86 *p* = 0.003), ASA grade (HR: 1.50 95% CI 1.15–1.95 *p* = 0.003) and SIG (HR: 1.28 95% CI 1.11–1.48 *p* < 0.001) remained independently associated with survival.

**Table 5:** The relationship between clinicopathological characteristics, tumour phenotyping, CT derived body composition and survival in patients undergoing surgery for colorectal cancer with T3 disease (*n* = 254): univariate survival analysis.

Characteristic			Univariate		Multivariate	
		*n* = 254	Overall survival HR (95% CI)	*P*-value	Overall survival HR (95% CI)	*P*-value
	Clinico-pathological					
Age	≤65	70 (27.6)	2.01 (1.63–2.49)	<0.001	1.45 (1.13–1.86)	0.003
	65–74	77 (30.3)				
	>74	107 (42.1)				
Sex	Female	108 (42.5)	1.16 (0.84–1.59)	0.364		
	Male	146 (57.5)				
ASA score (*n* = 217)	1	25 (11.5)	1.85 (1.47–2.33)	<0.001	1.50 (1.15–1.95)	0.003
	2	76 (35.0)				
	3	103 (47.5)				
	4	13 (6.0)				
Tumour location	Right	147 (57.9)	0.98 (0.72–1.33)	0.871		
	Left	107 (42.1)				
N-stage	0	163 (64.2)	1.16 (0.92–1.46)	0.209		
	1	69 (27.2)				
	2	22 (8.7)				
Ki67 (*n* = 223)	<30%	160 (71.7)	0.85 (0.57–1.26)	0.408		
	>30%	63 (28.3)				
Tumour budding (*n* = 244)	<10	178 (73.0)	1.32 (0.94–1.85)	0.114		
	>10	66 (27.0)				
Venous invasion	No	138 (54.3)	1.02 (0.75–1.39)	0.925		
	Yes	116 (45.7)				
Adjuvant chemotherapy	No	197 (77.6)	0.50 (0.33–0.76)	0.001		0.112
	Yes	57 (22.4)				
Margin involvement	No	251 (98.8)	2.20 (0.70–6.90)	0.178		
	Yes	3 (1.2)				
MMR status (*n* = 204)	dMMR	52 (20.9)	0.92 (0.72–1.18)	0.497		
	pMMR	152 (61.0)				
	MLH1/PMS2 or MHS 2/6	45 (18.1)				
GMS (*n* = 247)	0	43 (17.4)	1.52 (0.90–1.52)	0.255		
	1	160 (64.8)				
	2	44 (17.8)				
Necrosis	<10%	126 (49.6)	1.21 (1.01–1.45)	0.045		0.463
	10–30%	65 (25.6)				
	>30%	63 (24.8)				
	Systemic inflammation					
SIG	0	81 (31.9)	1.38 (1.22–1.57)	<0.001	1.28 (1.11–1.48)	<0.001
	1	64 (25.2)				
	2	61 (24.0)				
	3	31 (12.2)				
	4	17 (6.7)				
MUST (*n* = 76)	Low risk	61 (80.3)	2.29 (1.46–3.61)	<0.001		
	Medium risk	6 (7.9)				
	High risk	9 (11.8)				
	Body composition					
BMI (kg/m^2^) (*n* = 95)	≤25	29 (30.5)	1.01 (0.52–1.96)	0.966		
	>25	66 (69.5)				
High SFI (*n* = 95)	No	18 (18.9)	0.39 (0.20–0.76)	0.006		
	Yes	77 (51.1)				
Visceral obesity (*n* = 95)	No	24 (25.3)	0.64 (0.33–1.24)	0.188		
	Yes	71 (74.7)				
Low SMI (sarcopenia) (*n* = 95)						
SMI (Martin)	No	39 (41.1)	1.66 (0.85–3.22)	0.136		
	Yes	56 (58.9)				
SMI (Dolan BMI > 25)	No	36 (14.2)	2.14 (1.04–4.38)	0.038		
	Yes	59 (62.1)				
Low SMD (myosteatosis) (*n* = 79)						
SMD (Martin)	No	24 (30.4)	1.36 (0.60–3.06)	0.460		
	Yes	55 (69.6)				
SMD (Dolan BMI > 25)	No	29 (36.7)	1.61 (0.74–3.52)	0.234		
	Yes	50 (63.3)				
	Tumour phenotyping					

Single Hazard Ratios (HR) will be assigned to each variable in the table with individual categorical HR not being used.

*MMR* Mismatch Repair, *GMS* Glasgow Microenvironment Score, *SIG* Systemic Inflammatory Grade, *MUST* Malnutrition Universal Screening Tool, *BMI* Body Mass Index, *SFI* Subcutaneous Fat Index (subcutaneous obesity), *VFA* Visceral Fat Area (visceral obesity), *SMI* Skeletal Muscle Index (sarcopenia), *SMD* Skeletal Muscle Density (myosteatosis).

## Discussion

The results of the present study confirm the previously reported relationships between tumour necrosis and systemic inflammation, low muscle mass and survival, independent of T-stage. Multivariate analysis showed that age, ASA Grade and systemic inflammation were independent predictors of survival. Taken together the present results would suggest that tumour necrosis is intimately linked to downstream systemic effects including inflammation, loss of muscle mass and poor survival independent of disease stage. Indeed, there is recent evidence that cancer patients benefit from anti-inflammatory or immunomodulating medications [29, 30].

It has long been postulated that the combination of tumour necrosis and inflammation provides an environment in which the epigenetic regulation of genes, cell death, cell proliferation and mutagenesis occur [31]. At sites of chronic inflammation, cells are continuously dying as a consequence of hypoxic stress, an event in turn promoting growth and proliferation of the local epithelium. However, it was of interest in the present study that tumour necrosis was not significantly associated with tumour Ki-67, a marker of proliferation. Moreover, tumour necrosis was not significantly associated with vascular invasion. The present findings are consistent with previous work [32]. Therefore, the basis of tumour necrosis and its effects on the tumour microenvironment in patients with colorectal cancer are likely to be complex. Signalling pathways should be explored perhaps that are associated with hypoxia rather than inflammation. For example, HIF-1alpha and carbonic anhydrase IX (CAIX) may be a useful starting point to better understand the influence of tumour necrosis on the tumour microenvironment [33]. Indeed, there is some evidence that tumour necrosis was associated with over expression of CAIX and CAIX was an effective molecular marker of post chemoradiotherapy response in patients with rectal cancer [34].

In the present study the majority of patients had T3 disease and therefore further sub-analysis was carried out on patients with T3 disease to ascertain if the effects of tumour necrosis were independent of T-stage. However, this subanalysis may not prove that the effects of tumour necrosis are the same for other T-stages since tumour necrosis was lower in less advanced T-stages (Table 1). Therefore, it would be factually more correct to state that the relationship between tumour necrosis and systemic inflammation, low muscle mass and survival was independent of T3-stage disease. Larger cohorts are required to determine whether the above relationships also apply to other T-stages.

Nevertheless, given the above results tumour necrosis percentage has the potential to become an important clinicopathological factor in patients with colorectal cancer. In the present study 22% had extensive necrosis and 13% had extensive necrosis in the study of Richards and coworkers (2013) using a similar methodology and a percentage threshold of >30%, whereas in the study of Kastinen and coworkers [19] only 6% had extensive necrosis using a percentage threshold >40% [19, 35]. Therefore, there is need to standardize the guidelines for the measurement of tumour necrosis in colorectal cancer in a manner similar to that of consensus guidelines for tumour budding in colorectal cancer. This will enable the creation of a clinically valid, reproducible scoring system for tumour necrosis in patients with colorectal cancer. It may be that a reproducible tumour necrosis score will supplement the prognostic value of other prognostic pathological characteristics in the tumour such as venous invasion, inflammatory cell infiltrate, stroma percentage and budding. Furthermore, since the present study was carried out in primary operable colon cancer it would be of considerable interest to examine the present relationships in patients with metastatic disease.

The present study had a number of limitations inherent in retrospective analysis of cross-sectional studies (including prospective cohorts), principally sample bias. For example, although the patients were sequentially recruited the presence of an available preoperative CT scan for body composition analysis introduced a selection bias for body composition analysis (*n* = 181). Also, the measurement of tumour necrosis percentage was visually assessed that may results in some variability in the measured percentage tumour necrosis. Furthermore, some of the T-stage subgroups had very small n values and therefore such analysis would have been underpowered. However, there was a consistency between the present and previous results that suggest further work on tumour necrosis measurement in colon cancer is warranted.

In conclusion, tumour necrosis was associated with systemic inflammation, low muscle mass and survival, independent of age and T3-stage. This suggests a plausible connection between the tumour necrotic load and the systemic inflammatory response which may be a unifying explanation for skeletal muscle loss, reduced physical function and poorer outcomes. This suggests a more complex tumour/host interaction with tumour necrosis playing a part in driving systemic inflammation which in turn drives cachexia. Further work on the signal pathways underpinning the relationship between tumour necrosis and systemic inflammation is warranted and could provide new therapeutic targets for the treatment of cancer cachexia.

## Data Availability

Data will be made available upon requests to the corresponding author.
